# Development and Validation of an Algorithm for Segmentation of the Prostate and its Zones from Three-dimensional Transrectal Multiparametric Ultrasound Images

**DOI:** 10.1016/j.euros.2025.03.005

**Published:** 2025-04-06

**Authors:** Daniel L. van den Kroonenberg, Florian T. Delberghe, Auke Jager, Arnoud W. Postema, Harrie P. Beerlage, Wim Zwart, Massimo Mischi, Jorg R. Oddens

**Affiliations:** a Department of Urology, Amsterdam UMC, Amsterdam, The Netherlands; b Department of Electrical Engineering, Eindhoven University of Technology, Eindhoven, The Netherlands; c Department of Urology, Leiden University Medical Center, Leiden, The Netherlands; d Angiogenesis Analytics, JADS Venture Campus, ’s-Hertogenbosch, The Netherlands

**Keywords:** Segmentation, Prostate, Transrectal ultrasound, Artificial intelligence, Contrast-enhanced ultrasound, B mode

## Abstract

**Background and objective:**

Multiparametric ultrasound (mpUS) is being investigated as an alternative to magnetic resonance imaging (MRI) for detection of prostate cancer (PC). Automated prostate segmentation facilitates workflows, and zonal segmentation can aid in PC diagnosis, accounting for differences in imaging characteristics and tumor incidence. Our aim was to develop a deep learning algorithm that can automatically segment the prostate and its zones on three-dimensional (3D) contrast-enhanced ultrasound (CEUS) and conventional brightness-mode (B-mode) images (NCT04605276).

**Methods:**

A total of 259 3D mpUS images were collected from men with suspicion for PC in a prospective multicenter trial to develop a computer-aided diagnosis system for PC. Manual segmentation was performed using a custom tool, and an algorithm was developed using a convolutional neural network based on the U-Net architecture.

**Key findings and limitations:**

Cross-validation of the automated segmentation algorithm revealed Dice similarity coefficients (DSCs) of 0.91 (95% confidence interval [CI] 0.90–0.91) for CEUS and 0.94 (95% CI 0.93–0.94) for B-mode ultrasound for 3D prostate segmentation. Zonal segmentation was less accurate, with DSCs of 0.83 (95% CI 0.82–0.84) for CEUS and 0.86 (95% CI 0.85–0.87) for B-mode ultrasound. There was high agreement for prostate volume between automatic segmentation on CEUS and physician-estimated volumes on MRI (R^2^ = 0.96). Qualitative assessment of prostate segmentation using a scale from 1 to 5 revealed a median grade of 5 (interquartile range [IQR] 4–5) for manual segmentation and 4 (IQR 4–5) for automated segmentation (*p* = 0.10).

**Conclusions and clinical implications:**

Our deep learning algorithm demonstrated strong performance for automatic prostate and zonal segmentation from 3D CEUS and B-mode ultrasound images.

**Patient summary:**

We developed a computer tool to automatically identify the prostate in three-dimensional ultrasound images. The results show high accuracy and closely match manual assessments by urologists. This tool has potential for use in a computer-aided diagnostic system for prostate cancer.

## Take Home Message

We developed a deep learning algorithm for automated prostate and zonal segmentation of three-dimensional ultrasound images. This tool demonstrated high accuracy, closely matching expert assessments, and holds promise for improving prostate cancer diagnosis and streamlining imaging workflows in clinical practice.

## 1 Introduction

Imaging, especially magnetic resonance imaging (MRI), is playing an increasingly important role in the diagnosis of prostate cancer (PC). The clinical value of multiparametric ultrasound (mpUS) as a novel imaging modality for PC detection is being investigated as an alternative to MRI [1]. This new modality typically involves contrast-enhanced ultrasound (CEUS), shear-wave elastography, and brightness (B)-mode US [2]. Previous studies comparing mpUS to MRI have shown similar detection rates for clinically significant PC [3], [4]. Despite these promising results, current mpUS imaging methods are difficult to implement in clinical practice because of the dependence on operator expertise for acquisition and interpretation [5].

Computer-aided diagnosis (CADx) for PC based on mpUS could overcome the interobserver variability in interpretation currently associated with mpUS [1]. CADx algorithms have been facilitated by the recent availability of three-dimensional (3D) transrectal probes that allow reproducible quantitative US imaging. To support volume measurement, quantification, and feature selection, a CADx system requires accurate segmentation of the prostate and its zones, as these zones differ in tissue characteristics and PC prevalence [6]. However, manual segmentation is both time-consuming and prone to interobserver variability, making automated segmentation essential [7]. As mpUS CADx systems extract features from multiple US modalities, it is essential to be able to perform segmentation on both CEUS and B-mode images to ensure accurate spatial mapping and registration of the extracted features from both modalities.

Studies have shown that automated segmentation of the prostate boundaries and zones can be achieved with high accuracy for two-dimensional (2D) B-mode US [8]. Orlando et al [9] demonstrated the possibility of reconstructing 3D segmentation from 2D images. However, automated segmentation based on 3D CEUS has not yet been investigated. It is crucial to address this gap in order to advance CADx systems that can use the full capabilities of mpUS for PC detection.

Here we describe the development and validation of a deep learning algorithm to automate 3D segmentation of the prostate and its zones on CEUS and B-mode US images.

## 2 Patients and methods

### 2.1 Patient population

The patient population consisted of men undergoing radical prostatectomy or prostate biopsy and men with negative MRI findings (Prostate Imaging-Reporting and Data System score ≤2). These eligibility criteria were initially chosen to generate a data set for training a PC prediction algorithm based on mpUS as part of a CADx system (NCT04605276) [1]. The same cohort was subsequently used to develop and validate the segmentation algorithm in this study.

### 2.2 Transrectal 3D mpUS acquisition

The 3D mpUS images were acquired using a RIC5-9 3D end-fire probe (attached to a probe fixture) and a LOGIQ E10 ultrasound machine (GE Healthcare, Chicago, IL, USA; Supplementary material). The probe has an internal motorized mechanism for automated 120° volumetric sweeps [1]. The 3D mpUS imaging sequence consists of 3D B-mode and four-dimensional (4D) CEUS acquisitions. A single 3D B-mode sweep takes approximately 4 s, and the 4D CEUS acquisition lasts for ∼2 min. The mean CEUS intensity of each voxel is calculated over time to generate a 3D mean-intensity CEUS image. The specific scan settings can be found in the Supplementary material.

### 2.3 Manual image segmentation

Manual segmentation of 3D transrectal ultrasound (TRUS) images was performed by three authors (D.L.v.d.K., A.J., and A.W.P. with 2, 4, and 8 yr of TRUS experience, respectively). In cases of doubt, segmentation results were evaluated by a second operator. Segmentation was performed using a custom tool (Angiogenesis Analytics, Netherlands). The operator segmented the prostate by placing points on the boundary on the 3D B-mode image, as the boundary of the prostate is more distinct on B-mode US. A mesh was computed by constraining a smooth surface to the boundary points and was continuously updated as points were added. Finer adjustments could be made by locally dilating or eroding the mesh. Following prostate segmentation, the mesh was copied to the CEUS image, and the peripheral zone (PZ) and transition zone (TZ) were segmented using the same method [10]. The CEUS image is preferred to segment the zones as they are more easily distinguishable in this modality.

### 2.4 Automatic segmentation algorithm

**Fig. 1:**
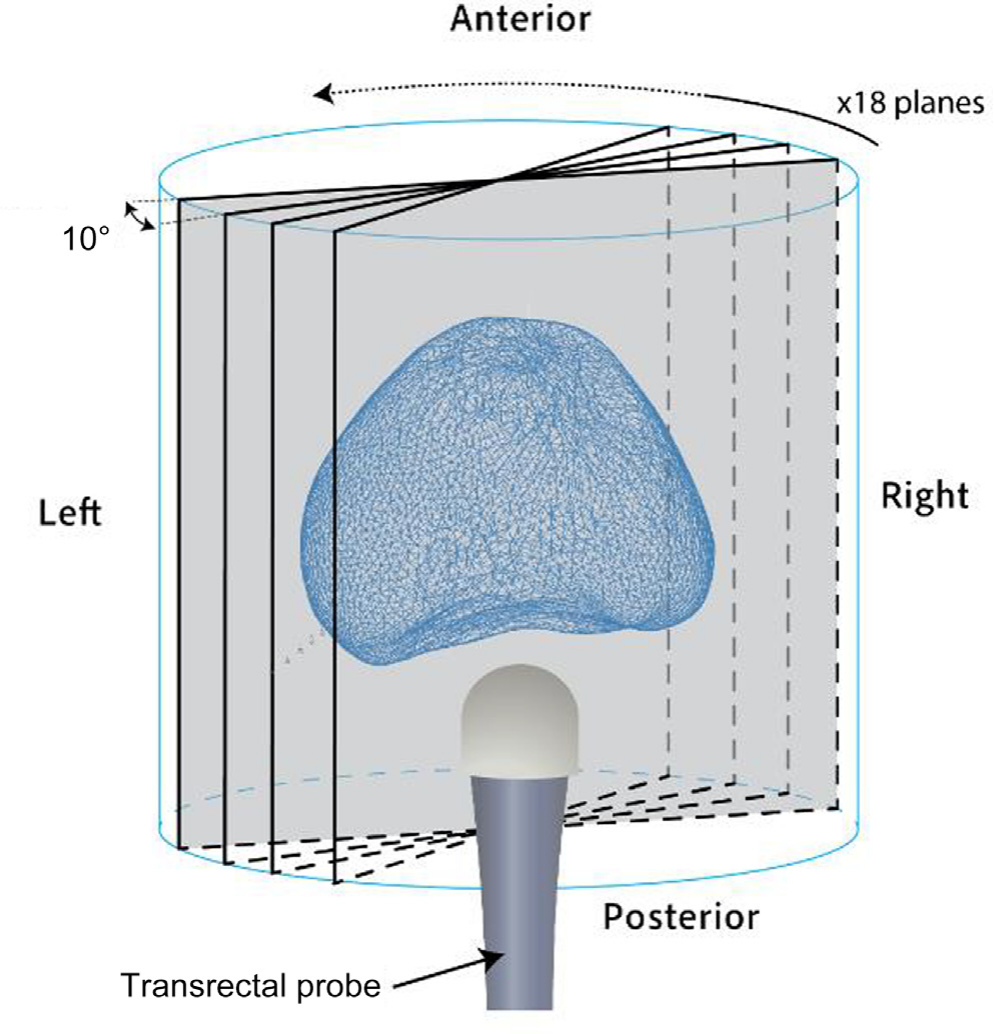
Radial sampling process with respect to the probe and prostate orientation. A total of 18 planes are extracted at 10° intervals, covering the whole cylinder.

For automated segmentation, the algorithm first resamples the 3D images into 2D radial planes (Fig. 1). This approach generates comparable views of the prostate in each plane, which facilitates more efficient learning [11].

In each plane, segmentation of the prostate and its zones was carried out using a convolutional neural network (NN) based on the U-Net architecture with 47 million trainable parameters [12]. Two NNs were trained, one for each US modality. Each NN has two output channels: the prostate location and the discrimination between zones. To improve consistency between subsequent planes, the NN also receives input from two additional parallel planes, with 4 mm between them.

Segmentation of the PZ is computed from the union of the prostate segmentation and the complement of the location of the TZ. The models were trained on 259 CEUS and 156 B-mode acquisitions, which aligns with similar studies [8], [9]. The training scheme used fivefold cross-validation and ran for 60 iterations, but stopped earlier if there were no further improvements in the validation metrics for ten iterations.

### 2.5 Quantitative analysis

Values for the Dice similarity coefficient (DSC) and intersection over union (IoU) are reported as measures of the overlap of the automatic and manual segmentation. Both metrics range from 0 (no overlap) to 1 (perfect overlap). DSC and IoU are similar as they report a ratio between the overlap of segmentation (predicted and ground truth) and the total segmented area, but the formulas differ (Supplementary material). DSC focuses on how well the prediction matches the ground truth, while IoU measures the overlap relative to the total area. Use of the two metrics provides a more complete evaluation and allows comparison with other studies. Finally, the relative volume difference, mean agreement (mean surface distance, MSD), and worst-case agreement (Hausdorff distance, HD) between the automated and manual segmentations were computed [13]. The values reported are the mean of predictions by each NN for its validation set and are in compliance with reporting guidelines (Supplementary material) [14]. Bland-Altman plots were used to compare automated prostate volumes with MRI-derived prostate volumes based on the ellipsoid formula [15]. We conducted this comparison because of the clinical utility of prostate volume in combination with prostate-specific antigen (PSA) for risk stratification. To calculate the computational time per segmentation, an 8-core 2021 MacBook Pro computer was used.

### 2.6 Qualitative analysis

To assess the clinical relevance and usability of automated segmentation beyond quantitative metrics, we conducted a qualitative evaluation. This approach provides a better understanding of how experts would evaluate these segmentations and their suitability for real-world clinical use. Median qualitative scores were compared for the manual and automated segmentation methods using a Wilcoxon signed-rank test.

Segmentations of the prostate and zones were overlaid on a CEUS image and shown to an expert with 3 yr of mpUS experience for grading on a scale from 1 to 5. Each image set contained six image planes from one prostate (Supplementary material). A sample of 51 prostates was analyzed, with an equal distribution of small (<40 cm^3^), medium (40–70 cm^3^), and large (>70 cm^3^) prostate volumes.

## 3 Results

### 3.1 Quantitative results

The proposed method resulted in accurate 2D segmentation of the prostate, with DSC values of 0.95 for B-mode and 0.93 for CEUS images (Table 1). Reconstruction of these segmentations to 3D versions led to a slight decrease in performance, except for HD, which improved. There was good agreement for prostate boundary between automated and manual segmentation: MSD remained <1 mm for B-mode US and <1.5 mm for CEUS. Each 3D segmentation was completed within a mean of 8 s for both the prostate and the zones.

**Table 1:** Comparison of automated and manual results for 2D and 3D segmentation of the prostate boundary and zones on B-mode and CEUS images^a^

Modality and task	IoU	DSC	RVD, %	MSD, mm	HD, mm
	(95% CI)	(95% CI)	(95% CI)	(95% CI)	(95% CI)
2D CEUS					
PB	0.87 (0.87–0.88)	0.93 (0.93–0.93)	–	1.98 (1.87–2.08)	6.08 (5.78–6.42)
Zones	0.80 (0.79–0.81)	0.88 (0.88–0.89)	–	2.56 (2.43–2.68)	7.59 (7.26–7.91)
2D B-mode					
PB	0.91 (0.90–0.91)	0.95 (0.95–0.95)	–	1.41 (1.33–1.49)	4.93 (4.62–5.29)
Zones	0.83 (0.82–0.84)	0.90 (0.90–0.91)	–	2.26 (2.14–2.37)	7.07 (6.75–7.38)
3D CEUS					
PB	0.83 (0.82–0.84)	0.91 (0.90–0.91)	7.3 (6.4–8.4)	1.33 (1.26–1.39)	6.09 (5.83–6.37)
Zones	0.72 (0.71–0.73)	0.83 (0.82–0.84)	17.2 (14.8–19.5)	1.77 (1.69–1.85)	6.92 (6.64–7.18)
3D B-mode					
PB	0.88 (0.87–0.89)	0.94 (0.93–0.94)	−0.4 (−1.7 to 0.8)	0.95 (0.89–1.02)	5.55 (5.17–5.97)
Zones	0.76 (0.74–0.77)	0.86 (0.85–0.87)	15.1 (12.4–18.5)	1.57 (1.48–1.67)	6.85 (6.45–7.24)

2D = two-dimensional; 3D = three-dimensional; CEUS = contrast-enhanced ultrasound; CI = confidence interval; IoU = intersection over union; DSC = Dice similarity coefficient; PB = prostate boundary; RVD = relative volume difference; MSD = mean surface distance; HD = Hausdorff distance.

a The values reported are the mean over all patients. The 2D results are the mean for all 18 slices.

Zonal segmentation showed lower performance than prostate segmentation across both imaging modalities. For CEUS, the DSC was 0.83 for zonal segmentation and 0.91 for prostate segmentation; the corresponding values for B-mode US were 0.86 and 0.94 (Table 1).

On B-mode US, prostate volumes closely matched between automatic and manual segmentation, with differences of <1%, while the TZ volume was 15% greater on automatic segmentation. However, mean CEUS-predicted values were 7% greater for prostate volume and 17% greater for TZ volume.

**Fig. 2:**
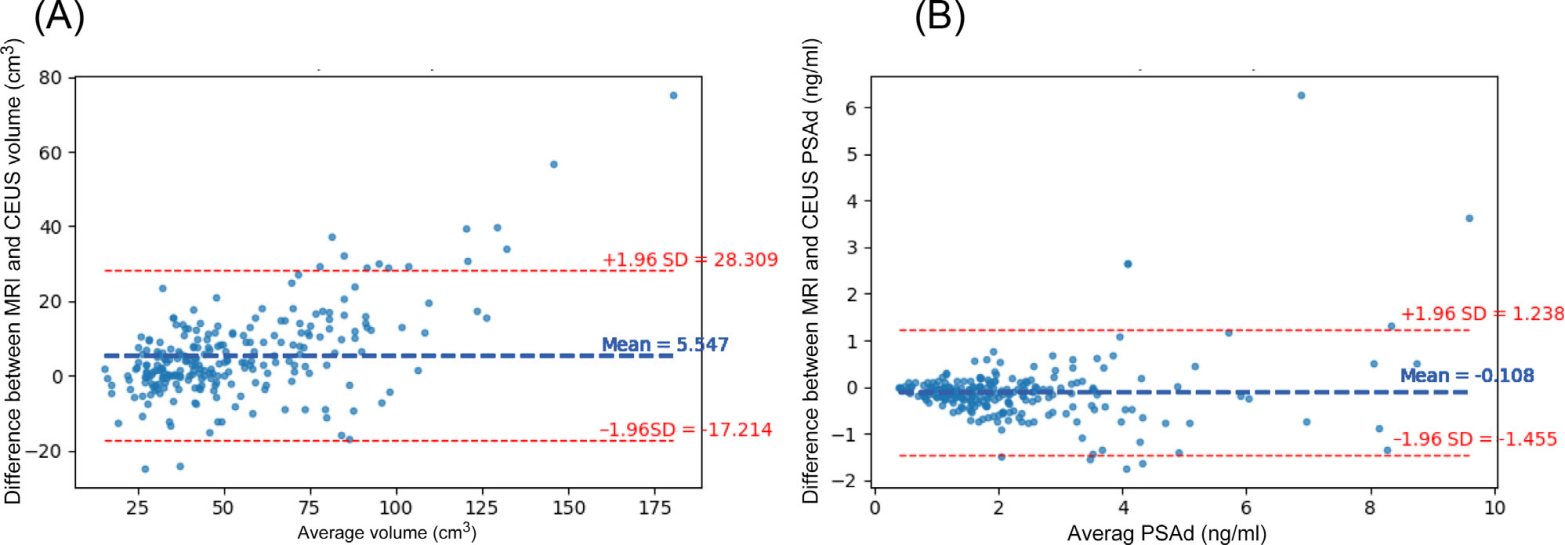
Bland-Altman plots showing the difference between magnetic resonance imaging (MRI) and contrast-enhanced ultrasound (CEUS) estimates of (A) prostate volume and (B) prostate-specific antigen density (PSAd) as a function of the average values. SD = standard deviation.

Prostate volume according to automatic segmentation on CEUS was also compared to MRI-derived results (Fig. 2). Agreement between volumes estimated using the two modalities was high (R^2^ = 0.96), but the mean volume measured on MRI was greater by 5.5 cm^3^ (*p* < 0.001). Median PSA density according to MRI-estimated prostate volume was 0.17 ng/ml/cm^3^ (interquartile range [IQR] 0.13), which is marginally lower than the result based on CEUS-estimated prostate volume (0.18 ng/ml, IQR 0.14; *p* < 0.001). Notably, the agreement between automated segmentation and MRI-estimated prostate volume was lower for larger prostates.

### 3.2 Qualitative results

The paired qualitative comparison involved evaluation of 51 prostates on six different planes by a single expert (D.L.v.d.K.). For prostate segmentation, the automated approach did not score lower than the manual approach, with median scores of 4 (IQR 4–5) and 5 (IQR 4–5), respectively (*p* = 0.10; Table 2). For zonal segmentation, the corresponding median scores were 4 (IQR 4–5) and 5 (IQR 4–5; *p* = 0.13). There were no differences in performance by prostate volume group.

**Table 2:** Grades for manual and automated segmentation according to qualitative expert analysis of the prostate and zonal boundaries

Grade	Segmentation, *n* (%)				Definition
	Manual		Automated		
	PB	Zones	PB	Zones	
5	30 (59)	31 (61)	22 (43)	24 (47)	Great: almost identical to the PB (up to 5-mm deviation)
4	16 (31)	15 (29)	24 (47)	22 (43)	Good: differs more evidently (>5-mm deviation allowed in the TZ); segmentation is larger than the real contour (ie, no tissue is missed)
3	5 (10)	5 (10)	5 (10)	5 (10)	Mediocre: differs evidently (>5mm) in the PZ; segmentation is smaller than the real boundary (ie, some tissue is missing)
2	0	0	0	0	Bad: Differs evidently (>5 mm) in multiple areas of the PZ
1	0	0	0	0	Unusable: does not match the PB

PB = prostate boundary; TZ = transition zone; PZ = peripheral zone.

## 4 Discussion

We developed a robust algorithm to segment the prostate and its zones in both B-mode and CEUS images. The algorithm uses deep learning to segment the prostate and zones in two dimensions, and the results are then reconstructed as a 3D segmentation.

The B-mode 2D results for prostate and zonal segmentation slightly outperform other studies [8]. Our B-mode 3D results are similar to those in other studies, but with a higher HD [9], [11], [16]. However, those studies did not include 3D segmentation of prostate zones, which is relevant for PC detection, MRI-TRUS registration, and biopsy targeting [8]. Our results indicate that zonal segmentation seems to be less accurate than prostate segmentation, as evident in the lower performance across all metrics. This observation aligns with feedback from urologists who have reported that the boundary between prostate zones is sometimes difficult to distinguish.

After 3D reconstruction, most metrics revealed lower agreement with the manual segmentation in comparison to 2D reconstruction, with greater discrepancy than that reported by Orlando et al [9]. The difference may be attributable to the authors’ use of the same 2D planes for 3D reconstruction of the images, the ground truth segmentation, and the automated segmentation [9]. By contrast, our approach involves direct manual segmentation on 3D images and automated segmentation of 2D planes for samples before reconstructing the 3D segmentations. The improvement in HD after 3D reconstruction is probably the result of a smoother segmentation boundary because of interpolation.

**Fig. 3:**
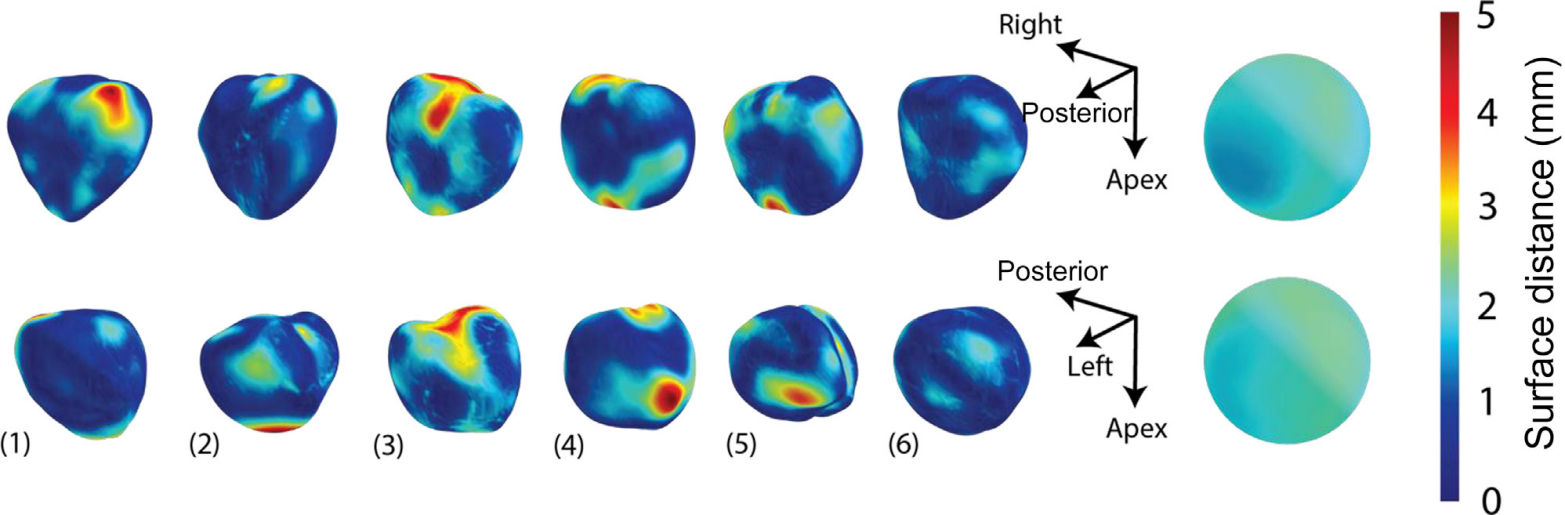
Six examples of prostate glands from two views with the predicted prostate boundary on contrast-enhanced ultrasound (CEUS). The color indicates distance to the manual segmentation at that point. The spheres on the right show the average surface distance for all 259 prostate samples projected onto a unit sphere (same scale).

We observed that the mean HD exceeded 6 mm. However, this affected only a limited part of the segmentation (Fig. 3). MSD between the two segmentations was consistently <2 mm. Clinically, this margin of error is acceptable, given that only suspicious lesions with a diameter of ≥10 mm (volume ≥0.5 cm^3^) are considered clinically significant [17]. Although this margin could theoretically result in a targeting error for smaller lesions, the clinical consequences of potentially missing such insignificant lesions remain uncertain because of their limited relevance. Moreover, this method tends to overestimate the size of the prostate, minimizing the risk of missing small lesions around the boundary of the gland.

The 3D plots suggest that automated CEUS segmentation slightly underperforms in the anterior area, but this effect is almost negligible, with MSD values <1 mm higher than for the posterior area (Fig. 3).

Automated segmentation had better performance on B-mode than on CEUS images. First, this could be because of tissue suppression in CEUS scans. This effect is more pronounced on the anterior side of the prostate and hence might alter automated CEUS segmentation in this area. Second, the acquisition time is 30 times shorter for B-mode US than for CEUS, making the latter more prone to patient movement and potentially resulting in less accurate segmentation. Both factors contribute to overestimation of the prostate volume on CEUS (Table 1). Despite these challenges, CEUS segmentation remains highly relevant for CADx using features based on the flow dynamics of the contrast agent [18].

PSA density is a valuable risk stratification tool for PC, but it requires accurate estimation of prostate volume. Our findings indicate a high degree of correlation for prostate volume between our automated segmentation and MRI estimates, confirming the utility of our proposed method for risk stratification. However, mean prostate volume was significantly higher according to MRI in comparison to automatic segmentation. Despite this difference, median PSA density values were comparable, with 0.17 ng/ml/cm^3^ for MRI and 0.18 ng/ml/cm^3^ for CEUS. Therefore, the slightly higher volume estimates had a limited impact on PSA density and risk stratification. Furthermore, it is important to note that the ellipsoid formula for MRI volume estimation is an approximation and does not fully capture the shape and actual volume of the prostate [15]. Consequently, mpUS estimation may prove to be more accurate than MRI-based estimation. However, the absence of a reference standard for prostate volume in these patients precluded an evaluation of which method is the most accurate.

Qualitative assessment of the practical usability and acceptability revealed that automated segmentation did not score significantly lower than manual segmentation. In addition, automated segmentation achieved a score of ≥4 out of 5 in more than 90% of cases, indicating that it can be used with little to no further adjustments. Furthermore, the improvement in efficiency, from approximately 10 min for manual segmentation to <8 s for automated segmentation, should be considered. Consequently, even with the need for occasional minor adjustments, automated segmentation represents a more efficient and consistent method than manual segmentation.

Our study has limitations that may affect the accuracy and reliability of automated segmentation, mostly in relation to the 3D reconstructions. According to most metrics, 3D reconstruction had lower performance than 2D segmentation. This decrease in performance can be attributed to several factors. First, the cylindrical interpolation method used for reconstruction may not accurately model the prostate anatomy and may lead to less accurate 3D reconstruction. In addition, the points used to define the prostate and zonal boundaries differ in number and position. This mismatch could lead to discretization errors, even if the surfaces are similar, and might result in lower performance of the 3D approach.

Although automated segmentation methods may have slightly lower accuracy than expert manual segmentation, they are expected to offer more consistent and reproducible results across different data sets and operators. Automated methods can streamline and accelerate the segmentation process and greatly reduce the effort required for manual segmentation by providing a good starting point. Our segmentation model could serve as a valuable source of information for a CADx algorithm for PC detection, especially because of differences in PC prevalence among different zones of the prostate.

## 5 Conclusions

Our deep learning algorithm for automatic segmentation based on CEUS and B-mode ultrasound reliably determines prostate volume, boundaries, and zones. The algorithm reduces the segmentation time and can thus alleviate the burden on physicians. Accurate segmentation is a critical component of CADx systems, and the performance of our algorithm underscores its potential to support future developments in automated PC detection.

  ***Author contributions:*** Daniel L. van den Kroonenberg had full access to all the data in the study and takes responsibility for the integrity of the data and the accuracy of the data analysis.

*Study concept and design*: van den Kroonenberg, Delberghe, Jager, Postema.

*Acquisition of data*: van den Kroonenberg, Delberghe, Jager, Postema.

*Analysis and interpretation of data*: van den Kroonenberg, Delberghe.

*Drafting of the manuscript*: van den Kroonenberg, Delberghe, Jager, Postema.

*Critical revision of the manuscript for important intellectual content*: van den Kroonenberg, Delberghe, Jager, Postema, Zwart, Mischi, Beerlage, Oddens.

*Statistical analysis*: van den Kroonenberg, Delberghe.

*Obtaining funding*: Mischi, Beerlage, Oddens.

*Administrative, technical, or material support*: None.

*Supervision*: Mischi, Oddens.

*Other*: None.

  ***Financial disclosures:*** Daniel L. van den Kroonenberg certifies that all conflicts of interest, including specific financial interests and relationships and affiliations relevant to the subject matter or materials discussed in the manuscript (eg, employment/affiliation, grants or funding, consultancies, honoraria, stock ownership or options, expert testimony, royalties, or patents filed, received, or pending), are the following: Auke Jager, Arnoud W. Postema, Massimo Mischi, and Harrie P. Beerlage are scientific advisors for Angiogenesis Analytics for which they receive compensation. Wim Zwart and Florian T. Delberghe are employees of Angiogenesis Analytics. Daniel L. van den Kroonenberg and Jorg R. Oddens have nothing to disclose.

  ***Funding/Support and role of the sponsor:*** Funding for this study was provided by Angiogenesis Analytics and the European Innovation Council Transition program under project #101057919 PCaVision. The sponsors played a role in data collection.

  ***Ethics considerations:*** This study was approved by an accredited medical research ethics committee (METC AMC) under reference number 2020_268#B202178. All study participants signed an informed consent form that included consent for use of their data for publication. The study was performed in accordance with the Declaration of Helsinki.

  ***Acknowledgments:*** We acknowledge the contributions of Anna Garrido Utrilla and Marije Zwart.

  ***Data sharing statement:*** The data sets used and/or analyzed during this study are available from the corresponding author on reasonable request.
