# Supplementary material for: Development and Validation of an Algorithm for Segmentation of the Prostate and its Zones from Three-dimensional Transrectal Multiparametric Ultrasound Images
Source: Eur Urol Open Sci. 2025 Apr 6;75:48–54. doi: 10.1016/j.euros.2025.03.005 (PMC12002784; doi:10.1016/j.euros.2025.03.005)
Supplement: Supplementary Data 1 [file mmc1.docx]

**Supplementary Material**

Table 1a: Ultrasound settings for CEUS

| **Setting** | **Value** |
| --- | --- |
| Axial voxel size (mm) | 0.14 – 0.17 |
| Dynamic range (db) | 42 |
| Gain (db) | 55 |
| Power level (%) | 10 |
| Transducer frequency (kHz) | 3500 |
| Mechanical index | 0.10 |
| Radius start (mm) | 12.389 – 15.2 |
| Radius mean step size (mm) | 0.14 – 0.17 |
| Radius min step size (mm) | 0.14 – 0.17 |
| Radius max step size (mm) | 0.14 – 0.17 |
| Radius jitter (mm) | 0.0 |
| Azimuth range (deg) | 110 – 150 |
| Azimuth mean step size (mm) | 0.79 – 0.81 |
| Azimuth min step size (mm) | 0.79 – 0.81 |
| Azimuth max step size (mm) | 0.79 – 0.81 |
| Azimuth jitter (mm) | 0.0 |
| Elevation range (deg) | 118 – 120 |
| Elevation mean step size (mm) | 2.30 – 2.372 |
| Elevation min step size (mm) | 2.30 – 2.372 |
| Elevation max step size (mm) | 2.30 – 2.372 |
| Elevation jitter (mm) | 0.0 |

Table 1b: Ultrasound settings for B-Mode

| Setting | Value |
| --- | --- |
| Axial voxel size (mm) | 0.05 – 0.14 |
| Dynamic range (db) | 69 |
| Gain (db) | 55 |
| Power level (%) | 100 |
| Transducer frequency (kHz) | 9000 |
| Mechanical index | 1.30 |
| Radius start (mm) | 12.389 – 15.2 |
| Radius mean step size (mm) | 0.05 – 0.14 |
| Radius min step size (mm) | 0.05 – 0.14 |
| Radius max step size (mm) | 0.05 – 0.14 |
| Radius jitter (mm) | 0.0 |
| Azimuth range (deg) | 110 – 150 |
| Azimuth mean step size (mm) | 0.30 – 0.32 |
| Azimuth min step size (mm) | 0.30 – 0.32 |
| Azimuth max step size (mm) | 0.30 – 0.32 |
| Azimuth jitter (mm) | 0.0 |
| Elevation range (deg) | 118 – 122 |
| Elevation mean step size (mm) | 0.70 – 0.72 |
| Elevation min step size (mm) | 0.70 – 0.72 |
| Elevation max step size (mm) | 0.70 – 0.72 |
| Elevation jitter (mm) | 0.0 |

|  | n = 259 |
| --- | --- |
| Age; median (IQR) | 69 (64 – 73) |
| Last serum PSA (ng/mL); median (IQR) | 8 (6.0 – 12) |
| Digital rectal exam (DRE)*; *N* (%) |  |
| Benign | 152 (60) |
| cT2 | 92 (37) |
| cT3/4 | 4 (1) |
| Missing | 4 (1) |
| MRI Prostate volume (ml); median (IQR) | 47 (36 – 74) |
| PSA density (median (IQR) | 0.16 (0.11 – 0.25) |
| MRI PI-RADS score; *N* (%) |  |
| PI-RADS ≤2 | 70 (28) |
| PI-RADS 3 | 13 (5.2) |
| PI-RADS 4 | 76 (30) |
| PI-RADS 5 | 92 (37) |
| No MRI | 1 |
| Radiological tumor stage; *N* (%)* |  |
| mT2 | 105 |
| mT3a | 48 |
| mT3b | 10 |
| mT4 | 0 |
| Not reported | 89 |
| ISUP grade from prostatectomy; *N* (%) |  |
| 1 | 2 |
| 2 | 85 |
| 3 | 71 |
| 4 | 1 |
| 5 | 21 |

Table 2: Patient characteristics

**Table 3**: Compliance with the TRIPOD+AI guidelines*.

| Title | Item # | Comment |
| --- | --- | --- |
| Title | 1 | In the title, the paper is identified as development and validation of a segmentation algorithm. |
| Abstract | 2 | The abstract follows the TRIPOD+AI for Abstracts guidelines |
| Background | 3a | The healthcare setting is currently addressed in the introduction |
|  | 3b | The targeted population is currently addressed in the introduction |
|  | 3c | This does not apply to our study, the study population is described in the supplementary results. |
| Objective | 4 | The objectives of the study are addressed in the introduction. |
| Data | 5a | The patient cohort is composed of patient participating in a prospective clinical trial for the development a computer-aided diagnosis tool to localize prostate cancer. Using cross validation, patients are randomly assigned to either the development or evaluation group retrospectively. The data is considered representative due to the inclusion of patients with and without prostate cancer. Further details concerning the cohort can be found in Table 2 of the supplementary material. |
|  | 5b | The patient data was acquired from June 2021 to February 2024. |
| Participants | 6a | The study was conducted at two academic hospitals in Amsterdam. |
|  | 6b | Patient eligibility criteria are addressed in the method section. |
|  | 6c | Patient treatment was described in the method section. |
| Data Preparation | 7 | The data from all patients was acquired using the same acquisition protocol. Each acquisition was manually checked for quality in terms of prostate visibility, motion artefacts and adequate contrast enhancement. |
| Outcome | 8a | The algorithm has been developed for the purpose of predicting the location of the prostate (zones) in B-mode and CEUS scans. The algorithm does not use any patient data relating to socioeconomic status. |
|  | 8b | The qualifications of the interpreter for the qualitative analysis are described in the method section. |
|  | 8c | The qualitative outcomes are blindly assessed, as described in the method section. |
| Predictors | 9a | The model predicts the prostate segmentation from one of the US acquisitions, this is the minimum set of available predictors. |
|  | 9b | Predictors are exclusively the gray-level values of the US images. Acquisition parameters are available in the supplementary materials. |
|  | 9c | Subjective predictors are not used in our study. |
| Sample size | 10 | The study size is constrained by the available dataset, initially acquired for a different purpose. The sample size was deemed adequate as it exceeds that of other studies in the literature concerning prostate segmentation. |
| Missing Data | 11 | Only complete data set was used in our study, incomplete data sets were excluded for this study. |
| Analytical Methods | 12a | Details to the cross-validation procedure are included in the Method section. The number of folds was optimized through a reduction in the size of the folds, with the increment in size of the training set being halted once no further performance enhancement was observed. |
|  | 12b | Images were normalized before input into the UNet model mapping [0, 255] gray-level range to [0, 1]. In addition, augmentations were used during training applied with a random probability. This included both optical (rescaling, shearing, mirroring) and noise (gaussian additive, salt and pepper) transforms. |
|  | 12c | The model used in this study is a UNet, a well-established approach for medical image segmentation. The architecture was based on the initial paper, with no modification made to the depth, sub-architecture of the blocks, or activation function. The number of parameters was optimized by adjusting the numbers of channels of the 2D convolutions. |
|  | 12d | Heterogeneity of the dataset was not estimated. |
|  | 12e | See the Methods section of supplementary materials for description of the metrics used and their justification. Bland-Altman plots were used to show that the performance of the model is not too affected by the size of the prostates. The surface distance and mean surface distance facilitate the reader to see that errors only occur in small areas and their overall distribution doesn't exhibit concerning trends or biases. |
|  | 12f | The authors did not observe any unexpected behaviors of the model requiring a recalibration or fine-tuning step. |
|  | 12g | The predictions are calculated using a sigmoid activation function to transform the score at the model's output layer into a probability of every location being in the prostate or its zones. The entire interface utilizes (proprietary) python command line scripts. |
| Class imbalance | 13 | The present study focuses on segmentation rather than classification; therefore, there is no class imbalance as all samples include a prostate. |
| Fairness | 14 | It was determined that no suspicious behavior was observed in the model, and thus further analysis of its fairness was deemed unnecessary. |
| Model output | 15 | The model predicts a probability map for the location of the prostate (or its zones). The segmentation was considered positive if the predicted probability of the location being within the prostate was higher than 0.5. |
| Training vs Evaluation | 16 | Due to the cross validation scheme there should be no significant difference between the training and evaluation groups on healthcare setting, eligibility criteria, outcomes or predictors. |
| Ethical Approval | 17 | The present study has been approved by the Ethical Committee of Amsterdam UMC, Amsterdam. |
| Funding | 18a | The present study was funded by Angiogenesis Analytics (‘s-Hertogenbosch, The Netherlands), who provided all materials required for acquisition and analysis. |
| Conflicts of Interest | 18b | As requested by European Urology Open Science, the manuscript described conflicts of interest. |
| Protocol | 18c | The protocol of the parent study is available and published (PMID: 36874606) |
| Registration | 18d | This study is registered at clinicaltrials.gov under NCT04605276. |
| Data Sharing | 18e | The data for this study is shared by Amsterdam UMC with Angiogenesis Analytics (‘s-Hertogenbosch, The Netherlands) under a data sharing agreement and is not publicly available. |
| Code Sharing | 18f | The code used for this study is owned by Angiogenesis Analytics (‘s-Hertogenbosch, The Netherlands) and is not publicly available. |
| Patient & Public Involvement | 19 | This study was conducted without patient or public involvement, as the research was of a technical nature. It was determined that such involvement would not have added value to the study. |
| Participants | 20a | The patients underwent a 3D mpUS scan prior to radical prostatectomy. Subsequent to this procedure, the scan was manually segmented. This process has been described in the method section. |
|  | 20b | Please see the Supplementary Material about patient characteristics |
|  | 20c | Due to the cross-validation scheme, the development and evaluation data are the same. |
| Model Development | 21 | The number of patients and outcome events are reported in the Methods section, including details of the cross-validation procedure. |
| Model Specification | 22 | The model is based on a UNet architecture, described in the Method section. Due to its proprietary nature, further details or freely accessible implementations are not available at this time. |
| Model Performance | 23a | The model’s performance is currently addressed in the Results. |
|  | 23b | The authors did not observe any heterogeneity in the evaluation. |
| Model Updating | 24 | No model updating was applied for this study. |
| Interpretation | 25 | Overall interpretation are currently addressed in the Discussion |
| Limitations | 26 | Limitations are currently addressed in the Discussion. |
| Usability in the context of current care | 27a | In case that data is of poor quality (i.e. shadowing, poor contrast enhancement), the accuracy of the algorithm could be affected. Users will be instructed to exercise caution when interpreting the model’s outputs. This is described in the Discussion section. |
|  | 27b | The algorithm requires intermediate coding knowledge to be used in its current state. It could be packaged in an intuitive application where less technical users can simply input a DICOM file and a segmentation of the prostate is outputted. |
|  | 27c | Future research steps are currently addressed in the Discussion. |

***Checklist template from:** Collins G S, Moons K G M, Dhiman P, Riley R D, Beam A L, Van Calster B et al. TRIPOD+AI statement: updated guidance for reporting clinical prediction models that use regression or machine learning methods BMJ 2024; 385 :e078378 doi:10.1136/bmj-2023-078378

**Supplementary Material**

Figure 1: Blind qualitative assessment prostate segmentation example


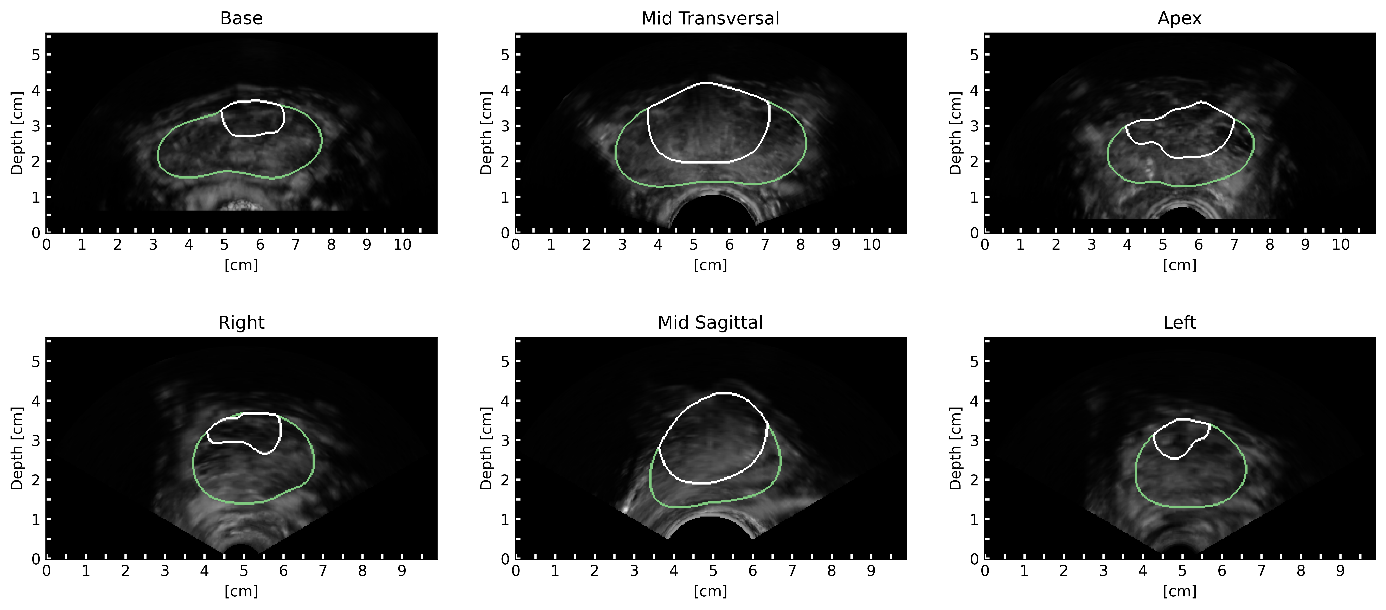


Figure 2**:** Example of automated and manual prostate and zonal segmentations for 4 patients. For each modality, the top row is the mid-sagittal plane, and the bottom row is the mid-transverse plane of the prostate.
**
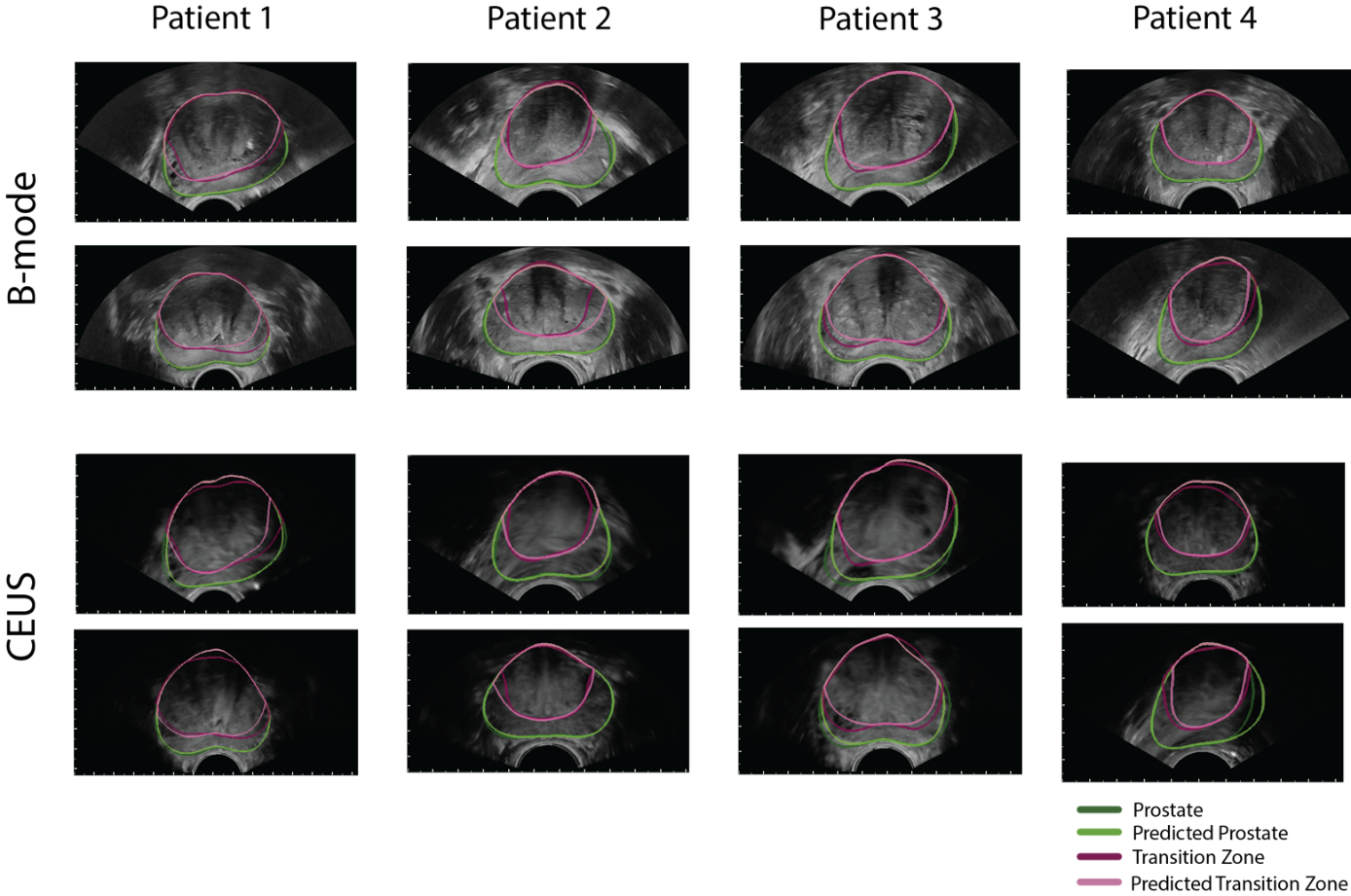
**

CEUS: contrast-enhanced ultrasound

Figure 3**:** Segmentation metrics

| **Dice Similarity Coefficient (DSC) =  2 * \|X ∩ Y\| / (\|X\| + \|Y\|)** | **Overlap between annotation X and annotation Y** |
| --- | --- |
| DSC = 2 * 0 / (1 + 1) = 0 | X = 1 X ∩ Y = 0 Y = 0 |
| DSC = 2 * 0.5 / (1 + 1) = 0.5 | X = 1 X ∩ Y = 0 Y = 0 |
| DSC = 2 * 1 / (1 + 1) = 1 | X = 1 X ∩ Y = 0 Y = 0 |

| **Intersection over Union** **(IoU) =  \|X ∩ Y\| / (\|X\| + \|Y\| - \|X ∩ Y\|)** | **Overlap between annotation X and annotation Y** |
| --- | --- |
| IoU = 0 / (1 + 1 - 0) = 0 | X = 1 X ∩ Y = 0 Y = 0 |
| IoU = 0.5 / (1 + 1 – 0.5) = 0.33 | X = 1 X ∩ Y = 0 Y = 0 |
| IoU = 1 / (1 + 1 - 1) = 1 | X = 1 X ∩ Y = 1 Y = 1 |

Haunsdorff distance (HD) =

Average

Mean Surface distance (MSD) =

Figure 4: Probe fixture
